# Developing mobile health applications for neglected tropical disease research

**DOI:** 10.1371/journal.pntd.0006791

**Published:** 2018-11-01

**Authors:** Andrés Navarro, Luisa Rubiano, Juan David Arango, Carlos A. Rojas, Neal Alexander, Nancy Gore Saravia, Eliah Aronoff-Spencer

**Affiliations:** 1 Universidad Icesi Grupo I2T, Cali, Colombia; 2 Centro Internacional de Entrenamiento e Investigaciones Médicas, CIDEIM, Cali, Colombia; 3 Universidad Icesi, Cali, Colombia; 4 Universidad de Antioquia, Medellín, Colombia; 5 University of California, San Diego, California, United States of America; Elisabeth Bruyere Research Institute, CANADA

## Abstract

Mobile applications (apps) can bring health research and its potential downstream benefits closer to underserved populations. Drawing on experience developing an app for detecting and referring cases of cutaneous leishmaniasis in Colombia, called Guaral/app, we review key steps in creating such mobile health (mHealth) tools. These require consideration of the sociotechnical context using methods such as systems analysis and human-centered design (HCD), predicated on engagement and iteration with all stakeholders. We emphasize usability and technical concerns and describe the interdependency of technical and human considerations for mHealth systems in rural communities.

## Introduction

Underserved rural communities in developing countries bear high burdens of poverty-related disease, with medical services often being impeded by logistical and economic barriers, displacement, and conflict. Mobile applications (apps) have the potential to overcome some of these barriers, enabling reliable and scalable point of care research and services.

Development of effective health research apps requires consideration of the sociotechnical context using methods such as systems analysis and human-centered design (HCD), predicated on problem refinement, engagement, and iteration with all stakeholders [1]. Besides patients, providers, and research teams, stakeholders may include public and private health authorities, caregivers, and communities at risk. Early stakeholder and end-user involvement is important and essential if the app is intended for use in health systems settings [2]. This communication considers challenges and requisites of mobile health (mHealth) tools based on lessons learned from developing Guaral/app, an app to be adopted by volunteer community workers for detection, and referral of patients with cutaneous leishmaniasis in rural Colombia [3].

To promote integration of the mHealth app into neglected tropical disease (NTD) research, we emphasize usability and technical concerns [4] and describe the interdependency of technical and human factors for mHealth systems in rural communities. Beginning with the statement of purpose, we consider user profiles, requirements, and then technology.

## Statement of purpose

The systems analysis process begins with the “statement of purpose” [5], which answers these questions: what is the goal of the app, and who will be the users? Answering these questions requires intensive, attentive, and methodical dialogue between engineers, health researchers, and users, oriented by the perception and needs of the community. For example, the purpose of the Guaral/app is to provide rapid, presumptive diagnosis of cutaneous leishmaniasis by nonprofessional health workers in or near affected communities. This purpose reflects a consensus among an underserved community (Tumaco), local health providers, and researchers to improve CL care and serves as a starting point both for iteration of the problem framing and ultimate solutions.

## User profiles

**Fig 1:**
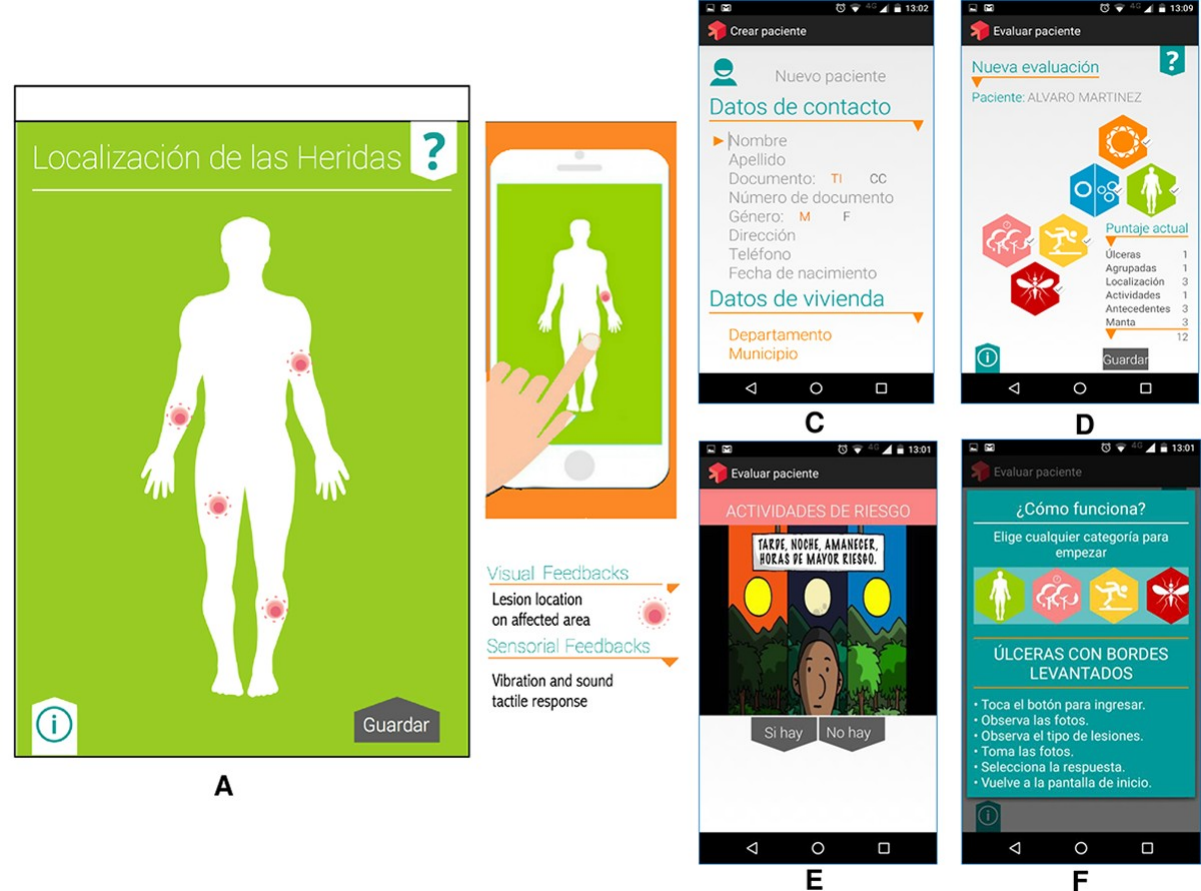
Screenshots of the mHealth tool (app) for presumptive diagnosis of leishmaniasis. Touch screen to register location of lesions (A, B); case identification data registry (C); intuitive icon menu of variables to be evaluated and automatic scoring screen (D); embedded video guide to the variables to be evaluated (E); tutorial on operation of the app (F).[3]. app, application; mHealth, mobile health.

App design must meet the needs of different users; these include the following:

- End users: If the end users include patients or other nonspecialists, who may perform and/or collect health data, particular attention is warranted for usability and interface design. In the case of the Guaral/app, the main end users are rural community health workers; therefore, an intuitive interface was developed and iterated to simplify interactions, such as recording of the location of patient lesions on a touch screen image of the body (Fig 1) while the system internally calculates the contribution of each variable to the diagnostic score.

- Principal investigator: The principal investigator (PI)’s interests—answering the research question—may have to be balanced with those of end users such as patients and health workers. In the leishmaniasis app, data capture is limited to a small set of variables, thereby facilitating use without compromising data quality.

## App requirements

The statement of purpose defines and frames the specific requirements of the app, which can be divided into two types (Box 1) [6,7]. Functional requirements are essential for the software to work, such as numeric calculations or adding a record to a database. Nonfunctional requirements refer to technical constraints such as asynchronous communications or regulatory needs, including data security [8].

**Box 1. Types of technical requirements in software development.**

**Functional**

These are functions that stakeholders expect the system to perform, e.g., register a new patient, assign patients to a doctor, or calculate drug dose. Other functions include data reports such as data outputs and visualization formats, e.g., infographics, statistical reports, and performance summaries.

**Nonfunctional**

Constraints may be imposed from diverse sources, including business logic or end users, who may expect a certain graphical user interface (GUI). Legal and regulatory norms, infrastructure, or security issues may also dictate requirements. For example, the use of expensive high-specification phones may be a risk in certain areas of social conflict, or the need to deactivate global position system (GPS). These constraints define requirements in terms of data security, audit, execution speed, capacity, availability, reliability, integrity, recovery, compatibility, maintainability, usability, and documentation.

In the case of Guaral/app, the core functional requirement is to deliver a presumptive diagnosis (positive or negative) of cutaneous leishmaniasis, based on answers to a few simple questions about clinical presentation and history. Nonfunctional requirements include operation without internet coverage.

## Security, privacy, and confidentiality

Security features are paramount to trust and acceptability and are considered from the planning stage [9]. If users or beneficiaries cannot trust an app, they will not use it or will not provide accurate information. Security involves two aspects of data management: (1) data capture, i.e., gathering and storing information in the mobile device, and (2) data transfer, i.e., transmission of information between devices, which requires technical specifications of data encryption and integrity [8]. Security features of the leishmaniasis app include strong encryption and two-factor authentication.

## Considerations in choosing technology

Choice of OS and development tools are key decisions.

### Operating system

The currently dominant OSs are Android and iOS. Android has the largest market share and is installed in devices with a wide range of prices and capabilities. Therefore, Android apps requiring limited functionality can use relatively inexpensive, expendable devices, whereas costlier devices are advantageous when advanced sensors such as an accelerometer or GPS are required. In comparison, iOS devices (iPhone and iPad) are more expensive yet most familiar to some types of users (Table 1).

**Table 1:** Technical considerations for mHealth apps.

Type of technology		Advantages	Limitations
Mobile apps, relative to older technologies such as paper and desktop computers		• Remote, synchronous and asynchronous mobile data collection • Automatically calculated variables and decision support • Decreased risk of human error • Faster interoperability with other electronic systems • Real-time monitoring and evaluation • Facilitated access to and by underserved populations • Replaces consumables such as paper and pens	• Poor performance in remote environmental conditions • Time and development for start-up • Difficulties in appropriation by end users • Stakeholders must acquire capacity or outsource app development and support • Poor compatibility with some other systems • Lack of infrastructure • Cost of technology • Risk of obsolescence
OS	Android	• Open source • Significant community support for development tools • Wide variety of prices and capabilities	• Security vulnerabilities • Lack of standardization because of multiple “flavors” of Android OS • More complex interactions and usability issues
	iOS	• Most familiar to some target users • Simple user interaction	• High device cost • Development is expensive
Development alternatives	Open-source customizable tools	• Rapidity of development • Lower learning complexity • Community support	• Lower level of customization • Greater need to train users • Database hosted on external servers
	Proprietary customizable and cross-platform development tools	• Standardized data management • More stable customizations • Attention to usability	• Development and functionality control may be limited or require significant skill to customize
	Tools developed from “scratch”	• Flexibility in design and development • Fully customizable • Highest user flexibility	• Complex development • Need for highly trained developers • Need for specialist usability designers • Significant debugging and need for updating and support
Approach	Interactive text messaging	• Lower cost of cellphone • Ease of use	Restrictions in the type of content (i.e., text or simple media only)
	Smart phone app	• Capture of more complex data, e.g., photos • Greater potential for automated decision support • GPS for localization	• Higher cost of data transmission • Complexity can affect usability

**Abbreviations**: app, application; GPS, global positioning system; mHealth, mobile health; OS, operating system.

### Development options

Selection of development options should consider factors such as user and developer sophistication, development time, and data requirements. Development strategies encompass (1) open customizable tools, including free and open-source software, (2) proprietary customizable tools, or (3) tools developed from scratch (Table 1).

The Android OS was chosen for the leishmaniasis app based on device cost, and the app was developed from “scratch” and tailored to end users using Android Studio, a free and widely used toolkit for mobile data collection.

## Discussion

The World Health Organization [2] has specified the requirements for health interventions using mobile apps. In particular, apps must have the functional suitability and usability to support the desired intervention and have stability, i.e., to operate reliably under field conditions with limited bandwidth or coverage interruptions. Additionally, mHealth apps should deliver the service specified by purpose (fidelity) and produce the expected results through its content and delivery (quality).

Internal and external contextual factors are critical to fulfilling these requirements and ultimately to implementation of the app. These are addressed through continuous stakeholder engagement and iteration based on measurable feedback. In the case of the leishmaniasis app, end users (community health workers and researchers) worked with software designers and engineers to iteratively conceive, co-design, develop, and evaluate prototypes of increasing functionality. Initial usability assessment has been positive and will be reported separately. The main challenges were limited connectivity and bandwidth, which delayed the data flow to the cloud, and thus to the researchers and physicians. Likewise, transport in the study area is mostly by motorized canoe, and one phone was lost in the river, although the data had already been uploaded to the cloud.

We have presented a brief examination of factors influencing the design, development, and deployment of mHealth apps for NTD research. Given that these diseases exist and must be studied within complex sociotechnical systems, we propose a road map for development based on systems thinking and HCD. The systems analysis approach for mHealth interventions and research begins with the understanding of contextual need and circumstances to determine purpose and then defines user profiles and app requirements within this context. A notable caveat to this approach, and a foundation of HCD, is that problem finding and profile development cannot be conducted appropriately without early and frequent stakeholder engagement and direct observations of users, in context, to develop accurate profiles and journey maps. Moreover, these must be refined as solutions are tested with users.

## Conclusion

Combining agile technology development (rapid iterations) and broad stakeholder commitment, particularly from local communities, can improve the development of interventions, optimize the evidence gathered, and accelerate the translation of knowledge into action.
